# New wave-type mechanism of saltatory conduction in myelinated axons and micro-saltatory conduction in C fibres

**DOI:** 10.1007/s00249-020-01442-z

**Published:** 2020-06-25

**Authors:** J. E. Jacak, W. A. Jacak

**Affiliations:** Department of Quantum Technology, Wrocław University of Science and Technology, 50-370 Wrocław, Poland

**Keywords:** Myelinated axon, Saltatory conduction, Ion plasmon-polaritons, Myelin role, Micro-saltatory conduction

## Abstract

We present a new wave-type model of saltatory conduction in myelinated axons. Poor conductivity in the neuron cytosol limits electrical current signal velocity according to cable theory, to 1–3 m/s, whereas saltatory conduction occurs with a velocity of 100–300 m/s. We propose a wave-type mechanism for saltatory conduction in the form of the kinetics of an ionic plasmon-polariton being the hybrid of the electro-magnetic wave and of the synchronized ionic plasma oscillations in myelinated segments along an axon. The model agrees with observations and allows for description of the regulatory role of myelin. It explains also the mechanism of conduction deficiency in demyelination syndromes such as multiple sclerosis. The recently observed micro-saltatory conduction in ultrathin unmyelinated C fibers with periodic ion gate clusters is also explained.

**Electronic supplementary material:**

The online version of this article (10.1007/s00249-020-01442-z) contains supplementary material, which is available to authorized users.

## Introduction

Na$^+$/K$^+$-ATPase plays a central role in the mechanism of action potential (AP) formation at nodes of Ranvier along myelinated axons (Debanne et al. 2011; Hodgkin and Huxley 1952). Sodium and potassium selective pores that span cell membranes function to conduct sodium and potassium ions down their electrochemical gradient, doing so both rapidly (up to the diffusion rate of these ions in bulk water) and selectively (despite the sub-angstroms difference in ionic radius). In neurons, the sodium channels are responsible for the rising phase of AP, whereas the delayed counterflow of potassium ions shapes the AP (FitzHugh 1961). Before an AP occurs, the axonal membrane is at its normal resting potential, ca. − 70 mV and Na$^+$ channels are in their deactivated state. In response to an increase of the membrane potential to about − 55 mV, the gates rapidly open, allowing sodium ions to flow into the neuron through the channels, and causing the voltage across the neuronal membrane to increase to $+$ 30 mV (in human neurons). Because the voltage across the membrane is initially negative, as its voltage increases from − 70 mV at rest to a maximum of $+$ 30 mV, it is said to depolarize. This increase in voltage constitutes the rising phase of an AP. The resting concentration of sodium ions inside a neuron cell is ca. 10 mM and ca. 140 mM outside, thus the opening of Na$^+$ channels in the neuron membrane causes a rapid diffusive inflow of these ions to the neuron cytosol according to the concentration gradient across the membrane. Next, with some time retardation inter-membrane channels for K$^+$ ions also open. The concentration of potassium ions at the rest state is opposite to sodium ions and equals ca. 140 mM inside a neuron cell and ca. 4 mM outside. Such a gradient of K$^+$ ions causes the diffusive influx of potassium through now opened K$^+$ channels across the membrane and the neuron repolarizes itself. The related decrease in voltage constitutes the falling phase of the AP. The formation of the AP spike can be mathematically modeled by the Hodgkin–Huxley (HH) formalism (Hodgkin and Huxley 1952) by a system of mutually coupled nonlinear differential equations that approximates the electrical characteristics of excitable neuron cells with electrically gated sodium and potassium channels across the cellular membrane. Due to the nonlinearity of the whole system the input signal to the block of ionic gates (as at the nodes of Ranvier along the myelinated axon) initiates the rapid increase of the *trans*-membrane electrical polarization which saturates at a constant its value shaping the AP spike. The input must be however larger than some threshold voltage, otherwise the response of the ion gate block does not occur (which follows from the Hopf bifurcation theorem for nonlinear differential equation systems). Such a behavior is called the all-or-none principle. Various modifications of HH models have been developed to include additional effects like e.g., leakage of ions due to the natural permeability of the membrane to ions beyond the voltage-gated channels, or in more detail, complex geometries of dendrites and axons, thermodynamic (Forrest 2014) and stochastic effects (Pakdaman et al. 2010) and others. Several simplified neuronal models have been also proposed (such as the FitzHugh–Nagumo model FitzHugh 1961; Nagumo et al. 1962), facilitating efficient large-scale simulation of groups of neurons, as well as mathematical insights into dynamics of action potential generation. In the HH model and its developed versions, both sodium and potassium channels are taken into account via coupled and mutually time retarded coupled nonlinear differential equations, which allow for the deriving of a perfect shape and size of the AP spike at the node of Ranvier on the time scale of 1 ms. On the longer time scale (of order even of 1 s) Na$^+$/K$^+$-ATPase restores the steady state of the Ranvier node. This latter phase of AP formation costs energy. For each ATP molecule a net single positive ion is transported outside the neuron to the surroundings restoring the steady polarization of the neuron membrane, − 70 mV.

Despite a realistic model for the AP spike formation at nodes of Ranvier (Hodgkin and Huxley 1952), the transduction of the igniting signal between consecutive nodes of Ranvier is not well described as of yet. The cable model [originated by William Thomson (the Lord Kelvin) to describe submarine cables (Thomson 1854)] of ion diffusion through the myelinated segment is ineffective because the upper limit of the velocity of this diffusion is by 1–2 orders of magnitude lower than the observed velocity. The cable model describes well the slow charge kinetics in dendrites (Rall et al. 1992; Rall 1989) and in nonmyelinated axons (Rall 1977; Jack et al. 1983; Cooley and Dodge 1966; FitzHugh 1973), being typically lower than 1 m/s, whereas the signal velocity in myelinated axons reaches 100–300 m/s, and reducing it by even only 10% prevents functionality of an organism. The mechanism behind these kinetics is apparently beyond diffusion transport described in cable theory and is referred to as saltatory conduction because transduction of the initial signal between nodes of Ranvier resembles almost instant saltation of the voltage along the myelinated segment, something absolutely unreachable for ordinary diffusion of ions according to cable theory. Moreover, there is evidence that the saltatory conduction has a wave type character. This conduction is maintained even if several nodes of Ranvier are damaged or the axon is broken into two parts with ends separated by a small break. This absolutely precludes the diffusion type current conduction because it is impossible for this to be maintained despite the break and indicates that the saltatory conduction must be of a wave type nature, which allows for jumping across small breaks and continuation of the travel despite damage and inactivation of several nodes of Ranvier (Debanne et al. 2011).

We propose a new model of saltatory conduction in the form of ionic plasmon-polariton dynamics, i.e., of collective synchronized ion oscillations in myelinated segments propagating as a wave along the periodically myelinated axon. The model takes advantage of the ion analogue to electron plasmon-polaritons in metallic linear periodic systems (Pitarke et al. 2007; Citrin 2004). Plasmons are collective fluctuations of the charge distribution, in metals—of mobile electron clouds (Maier 2007), in liquid electrolytes—of ion clouds (Jacak 2015b). In equilibrium states, the metals or electrolytes are neutral due to a perfect balance of opposite sign charges, but in excited states, some local polarization of charge clouds may oscillate and these oscillations are called plasmons (Pines 1999). A uniform movement of one sign charges with respect to oppositely signed ones results in surface plasmons in the case of finite systems (metallic particles or electrolytes confined by membranes) where imbalanced charge fluctuations occur only on the system boundaries (Maier 2007). Surface plasmons have a lower frequency (lower energy) than their volume nonuniform counterparts and in noble metallic nanoparticles the surface plasmon frequencies are located in the range of visible light (Maier 2007; Barnes et al. 2003). For confined electrolytes the frequency of ionic plasmons can be much lower because of ca. $10^4$ times larger masses of ions than of electrons and orders of magnitude lower ion concentration in electrolytes in comparison to electrons in metals (Jacak 2015b). The lower energy of ionic plasmons may fit with the energy scale of living bio-matter and might play a role in signaling at the cellular level of bio-matter organization. Of particular interest would be so-called plasmon-polaritons, which are synchronized surface plasmon oscillations in periodic arrangements of finite plasmonic systems with mobile charges (Barnes et al. 2003; Brongersma et al. 2000). Surface plasmon oscillations in each segment of their linear alignment, let us say of a chain of metallic nanoparticles or of a chain of ionic systems, interact between themselves mostly due to dipole interaction via the electromagnetic (e-m) field induced by local dipoles of plasmons oscillating in segments. As a result, a coherent plasmon oscillation arises in all segments synchronized by their mutual e-m interaction, which propagates as a wave along a chain of plasmonic elements. This wave can transmit the energy and information and is called the plasmon-polariton (Pitarke et al. 2007; Berini 2009). No net electrical current is carried by plasmon-polariton waves but the transmission of a signal (as of a voltage of the local plasmon polarization) is similar as for ordinary electrical current. The group velocity of wave packets of plasmon-polaritons exceeds usually the velocity of carriers in ordinary charge currents. Thus the communication by plasmon-polaritons in periodic alignments of plasmonic segments will be more effective and quicker in comparison to diffusion-like charge currents.

The periodicity of a linear alignment of plasmonic segments is an unavoidable prerequisite for the organization of the plasmon-polariton dynamics. Therefore, the periodically myelinated axons with segments separated by small nodes of Ranvier are good candidates for the plasmon-polariton communication. In the present paper we will describe and analyze ionic plasmon-polariton kinetics in such neurons in order to explain the very quick so-called saltatory conduction in myelinated axons in a development of former ideas from the author (Jacak 2015c). The supporting argument for a new concept of signaling in periodically corrugated axons via plasmon-polariton propagation has arisen recently due to the discovery of periodicity in the distribution of ion gates along nonmyelinated thin C-fibers of peripheral axons transmitting nociceptive pain sensations (Neishabouri and Faisal 2014). The clustering of Na$^+$ channels on lipid rafts periodically distributed along C-fibers resembles the structure of nodes of Ranvier in myelinated axons and also allows for plasmon-polariton communication with high speed, which can explain the quick and effective so-called micro-saltatory conduction in C-fibers.

The subject of this paper meets with a recent large increase in interest and advances in plasmonics and of plasmon-polariton kinetics in metallic nano-systems and astonishing opportunities for subdiffraction light manipulations by plasmon excitations in nanoscale metallic components (Barnes et al. 2003), which caused the related rapid development of the new field of nanoplasmonics that overlaps with many possible applications of nanophotonics and sub-diffraction opto-electronics (Brongersma et al. 2000; Maier 2007; de Abajo 2010; Barnes et al. 2003; Pitarke et al. 2007; Berini 2009).

Plasmons in metals correspond to oscillations of local charge density of electrons with respect to positive jellium. This effect is quantum in nature, because the coherent movement of all charge carriers is conditioned by their mutual repulsion and cannot be understood classically. The successful quantum description of this phenomenon has been provided by Pines (1999) in the form of a random phase approximation (RPA) approach. A similar plasmon effect concerns also electrolytes with charge carriers in the form of ions instead of electrons (Jacak 2015b). Ca. $10^4$ times larger masses of ions in comparison to electrons and the concentration of ions in electrolytes much lower than of electrons in metals highly reduce the energy of ion plasmons to a scale which fits the energy scale of bio-systems. Similarly, the plasmonic size featured by a strong radiation of plasmons is shifted from the nano-scale for electrons to micrometer scale for ions, again just as for the scale of bio-cell organization. The RPA model of ion plasmons has been developed (Jacak 2015b) including the application to synchronized plasmon oscillations in linear periodic alignments of electrolytes confined by membranes (Jacak 2015c) in analogy to plasmon-polaritons in metallic nano-chains (Brongersma et al. 2000; Maier and Atwater 2005; Pitarke et al. 2007; Huidobro et al. 2010; Citrin 2004; Jacak 2013, 2014; Jacak et al. 2015).

The paper is organized as follows. In the next paragraph, the insufficiency of the cable theory to explain the saltatory conduction in myelinated axons is demonstrated. Next, the main idea of the new concept for the saltatory conduction in the form of wave type propagation of ionic synchronized plasmon oscillations, called plasmon-polaritons, in myelinated segments of an axon is presented. This paragraph is followed by the rigorous mathematical formulation of plasmon-polariton dynamics in linear alignments of a confined electrolyte system and the velocity of an ionic plasmon-polariton is derived. Next the fitting of the physical model to real chemical parameters and the geometry of neurons is analyzed. In the following paragraph the role of the thickness of the myelin sheath is considered and a suitable model for a control mechanism over the saltatory conduction is demonstrated. Finally, the model developed for a wave type propagation of the signal along the periodic structure of C-axons, very thin and long unmyelinated peripheral axons responsible for transmitting nociceptive pain sensations, is proposed upon the plasmon-polariton model of the related micro-saltatory conduction in good agreement with recent experimental observations. The new wave type model of neuron signaling in periodic corrugated axons is placed in the context of real neuronal system behavior both in the central and the peripheral human nervous systems. A review of the conventional cable theory model as a limiting diffusive regime for lengthy transmission that neglects inductance given in the Supplementary Information.

## Insufficiency of the cable theory to explain the saltatory conduction

Signal kinetics in dendrites or unmyelinated axons is well described by cable theory (Ermentrout and Terman 2010; Dayan and Abbott 2001; Izhikevich 2007) (for short derivation cf. Supplementary Information). Upon this theory the velocity of a signal is characterized by $v_c= \frac{\lambda }{\tau }=\frac{\sqrt{G}}{C\sqrt{R}}$, where *C* is the capacity across the neuron cell membrane per unit of the axon length, *G* is the conductance across the membrane and *R* is the longitudinal resistance of the inner cytosol, both per unit of the neuron filament length, and $\lambda$ and $\tau$ are space and time diffusion ranges defined in the cable model (Ermentrout and Terman 2010; Dayan and Abbott 2001; Izhikevich 2007; Debanne et al. 2011; Thomson 1854; Brzychczy and Poznański 2011). The velocity $v_c$ scales as $\sqrt{d}$ with the dendrite (axon) diameter, *d*, because $C\simeq \frac{\varepsilon _0 \varepsilon \pi d}{\delta }$, $1/G\simeq \frac{\rho _1\delta }{\pi d}$ and $R\simeq \frac{4\rho }{\pi d^2}$, where $\delta$ is the cell membrane thickness, $\rho$ is the longitudinal resistivity of the inner cytosol and $\rho _1$ is the resistivity across the membrane. For example values of the membrane capacity per surface unit, $c_m =1$ $\upmu$F/cm$^2$, $\rho _1 \delta =20{,}000 \;\Omega$ cm$^2$, $\rho = 100\;\Omega$ cm and the diameter of the cable $d= 2\;\upmu$m, one gets $v_c\simeq 5$ cm/s (Ermentrout and Terman 2010).

Due to presence of a myelin sheath in myelinated axons, both the capacity and conductance across the myelinated membrane are reduced, roughly inversely proportional to the myelin layer thickness. Thus, the cable theory velocity, $\sim \frac{\sqrt{G}}{C\sqrt{R}}$, grows ca. 10 times if the capacitance and conductance lower ca. 100 times. This is, however, still too low to match observations of kinetics in myelinated axons (Ermentrout and Terman 2010; Dayan and Abbott 2001; Izhikevich 2007; Scurfield and Latimer 2018; Fribance et al. 2016; Richardson et al. 2000; Waxman and Bennett 1972; Goldman and Albus 1968; Moore et al. 1978; Debanne et al. 2011; Song et al. 2019). Moreover, the activity of nodes of Ranvier slows down the overall velocity of the discrete diffusion in the myelinated internodal segments to the level of factor 6 instead of 10 (Ermentrout and Terman 2010). For realistic axons with $d\sim 1\;\upmu$m the assessed cable theory velocity gives ca. 3 m/s instead of 100 m/s observed in such-size myelinated axons in the peripheral human nervous system (PNS). A similar inconsistency in the cable model estimations is encountered in human central nervous system (CNS) with thinner myelinated axons of $d\in (0.2,1)\,\upmu$m (Scurfield and Latimer 2018).

To model larger velocities upon the cable theory accommodated to myelinated axons, much thicker axons are assumed with longer internodal distances, because upon the discrete diffusion model (Ermentrout and Terman 2010), the velocity in a myelinated axon, $v_m\simeq \sqrt{\frac{l}{d_0}}v_c$, where *l* is the length of internodal segments and $d_0$ is the length of the node of Ranvier. For instance, in Scurfield and Latimer (2018) and Richardson et al. (2000) $d_0=10\,\upmu$m (and greater) and $l=1150\,\upmu$m (and greater) have been assumed to gain the velocity of the AP transduction, $v_m\sim 40$ m/s (cf. also Waxman and Bennett 1972; Goldman and Albus 1968; Moore et al. 1978; Song et al. 2019), i.e., they are ca. ten times larger in dimension than the actual myelinated axons of a human. It is thus clear that the cable model is not effective in modeling the observed quick saltatory conduction in myelinated axons (Debanne et al. 2011; Richardson et al. 2000; Keener and Sneyd 2009; Izhikevich 2007; Scurfield and Latimer 2018; Dayan and Abbott 2001; Fribance et al. 2016; Waxman and Bennett 1972; Goldman and Albus 1968; Moore et al. 1978; Song et al. 2019). The velocity predicted upon the discrete cable model for realistic axon parameters is by at least one order of magnitude smaller than observed.

Combining the Huxley–Hodgkin (HH) mechanism at nodes of Ranvier with the cable model diffusion at internodal myelinated segments results in the estimation of the AP propagation velocity (Waxman and Bennett 1972; Moore et al. 1978) only ca. six time greater than in unmyelinated axons with the same geometry (Ermentrout and Terman 2010; Dayan and Abbott 2001; Izhikevich 2007), despite the reducing of the intercellular capacity and conductivity by the myelin sheath. The simplified formula for this velocity mentioned above (for iterative discrete diffusion model Ermentrout and Terman 2010) gives $v_m\sim \sqrt{\frac{l}{d_0}}$, and since $d_0$ is often 1 $\upmu$m and *l* is around 100 $\upmu$m, the increase in velocity of myelinated axons can be almost 10 times that of unmyelinated axons [more precisely the factor is closer to 6 (Ermentrout and Terman 2010)].

The velocity of the AP transduction must maintain a high value because deviation by 10% end bodily function (Scurfield and Latimer 2018). Continued mathematical attempts to optimize a model of the HH mechanism mixed with cable theory (Waxman and Bennett 1972; Moore et al. 1978; Ermentrout and Terman 2010; Dayan and Abbott 2001; Izhikevich 2007) in order to obtain a sufficiently high AP velocity in myelinated axons has not been successful over a long time, which strongly suggests that the way to understand the saltatory conduction must be linked with a different physical mechanism rather than any version of the cable theory of ion diffusion.

The cable theory is in fact the conventional model of a line of transmission widely applied in electronics and communication science. The derivation of the cable model is presented in Supplementary Information.

The diffusion of ions according to cable theory is too slow to explain the rapid saltatory conduction along myelinated segments of axons. Apparently, a different mechanism for this conduction is required beyond local diffusion. We propose such a new approach by synchronized oscillations of local ion density, which can propagate along the periodically myelinated axon in the form of a wave plasmon-polariton, very well-known from the similar phenomenon in metallic periodic linear systems. The mathematical model of a plasmon polariton will be described in the following section based on the analysis of local oscillations of ion density, called ion plasmons. The mathematical model of ionic plasmons is presented in Jacak (2015b).

In the discrete diffusion model (Ermentrout and Terman 2010) or in other modifications of the cable theory (Dayan and Abbott 2001; Izhikevich 2007) the HH cycle is included as the integrative element of local electricity in the closest adjacent myelinated segments. In the plasmon-polariton model, the HH cycles at consecutive nodes of Ranvier are decoupled from the synchronic oscillations of ion density in myelinated segments. The HH cycles are triggered by the plasmon-polariton wave packet propagating along the axon with high speed. HH cycles play, however, a role in control over the plasmon-polariton via the resonance selection of particular modes which are supplemented in energy and thus traversing arbitrary long distances with constant amplitude despite large damping and Ohmic losses in ion oscillations.

## Plasmon-polaritons in a chain of finite ionic systems—model of the saltatory conduction in myelinated axons

To solve the problem of explaining saltatory conduction, we develop a new model for it based on the kinetic properties of collective plasmon-polariton modes propagating along linear and periodically modified electrolyte systems, which for an axon is the thin cord of the nerve cell periodically wrapped by Schwann cells creating a periodic and relatively thick myelin sheath [Schwann cells myelinate axons in the PNS, whereas in the CNS, axons are myelinated by oligodendrocytes (Lazarevich and Kazantsev 2013; Debanne et al. 2011)]. The plasmon-polaritons were investigated and understood based on the well-developed domain of plasmonics (Barnes et al. 2003; Pitarke et al. 2007), especially nanoplasmonics applied to long-range low-damped propagation of plasmon-polaritons along metallic nanochains (Maier and Atwater 2005). The main properties of these collective excitations occurring on the conductor/insulator interface (Zayats et al. 2005; de Abajo 2010) due to the hybridization of the surface plasmons (i.e., the charge density fluctuations on the conductor surface) with the electromagnetic wave are as follows: (1) much lower velocity of plasmon-polaritons than the velocity of light, which yields plasmon-polaritons with wavelengths much shorter than the wavelength of light at the same frequency, (2) the related strong discrepancy between the momenta of plasmon-polaritons and photons with the same energies causes the external electromagnetic waves to not interact with plasmon-polaritons, i.e., photons cannot be excited or absorbed by plasmon-polaritons due to momentum conservation constraints, (3) all the electromagnetic field associated with propagation of plasmon-polaritons is compressed to the tunnel-volume of the chain, (4) all the radiative losses are quenched, and plasmon-polariton attenuation occurs due to the Ohmic losses of oscillating charged carriers, which makes periodically corrugated conductors almost perfect waveguides for plasmon-polaritons, and (5) long-range and practically undamped propagation of plasmon-polaritons is experimentally observed in metallic nanochains (Maier and Atwater 2005; Brongersma et al. 2000). The plasmon-polaritons in metallic nanostructures are likely to be exploited for future applications in optoelectronics where conversion of light signals into plasmon-polariton signals circumvents diffraction constraints that greatly limit the miniaturization of conventional optoelectronic devices (as the nanoscale of electron confinement inconveniently conflicts with the several-orders-of-magnitude larger scale of the wavelength of light at an energy similar to the nanoconfined electrons) (de Abajo 2010; Citrin 2005).

All properties of plasmon-polaritons can be repeated in periodic linear arrangements of electrolyte systems with ions instead of electrons as charge carriers (Jacak 2015b, c). According to the larger mass of ions compared with that of electrons and the lower concentration of ions in electrolytes compared with the concentration of electrons in metals, the plasmon resonances in finite ionic systems (e.g., liquid electrolyte confined to a finite volume by appropriately formed membranes, frequently found in biological cell structures) occur on the scale of micrometers rather than nanometers such as for metals and at frequencies (energy) several orders of magnitude lower (depending on the ion concentration).

For a spherical electrolyte system, the surface and volume plasmons are handled analogously to those of the metallic nanosphere as developed in Jacak (2015b). The ionic surface plasmon frequencies are given for the multipole *l*th mode by the formula $\omega _l=\omega _p \sqrt{\frac{l}{\varepsilon (2l+1)}}$ with the bulk plasmon frequency $\omega _p=\sqrt{\frac{4 \pi q^2 n}{m}}$ (*n* is the ion concentration, *q* and *m* are the ion charge and mass, respectively, and $\varepsilon$ is the dielectric relative permittivity of the surroundings). For dipole surface plasmons ($l=1$), this equation resolves to the Mie-type formula (Mie 1908; Jacak et al. 2010) $\omega _1=\frac{\omega _p}{\sqrt{3 \varepsilon }}$. The plasmon oscillations intensively radiate their own energy and are quickly damped due to Lorentz friction losses (i.e., due to radiation of e-m waves by oscillating charges (Landau and Lifshitz 1973; Jackson 1998)), which for large systems with a large number of ions participating in the plasmon oscillations (thus strengthening the Lorentz friction) are much greater than the Joule-heat dissipation caused by Ohmic losses due to carrier scattering (scattering of ions on other ions, solvent and admixture atoms and the boundary of the system) (Jacak 2015a, b).

Surprisingly, for a linear chain of spherical ionic systems in a dielectric surroundings, the radiation losses are completely reduced to zero in exactly the same manner as in metallic chains (Citrin 2006; Markel and Sarychev 2007; Jacak 2013). The radiation energy losses expressed by the Lorentz friction (Landau and Lifshitz 1973; Jackson 1998) are ideally compensated by the income of the energy due to the radiation from all the other spheres in the chain. As a result, the radiative losses are ideally balanced, and only the relatively small irreversible Ohmic energy dissipation remains due to ion scattering. Thus, the collective surface dipole plasmon-polaritons can propagate in the chain with strongly reduced damping, and if the energy is permanently supplemented to balance the small Ohmic losses, this propagation can occur over arbitrarily long distances without any damping. However, if the ionic chain is embedded in an absorbing medium, like in another electrolyte, then strong damping of plasmon-polaritons occurs.

### Plasmon-polariton propagation in linear periodic ionic systems

**Fig. 1:**
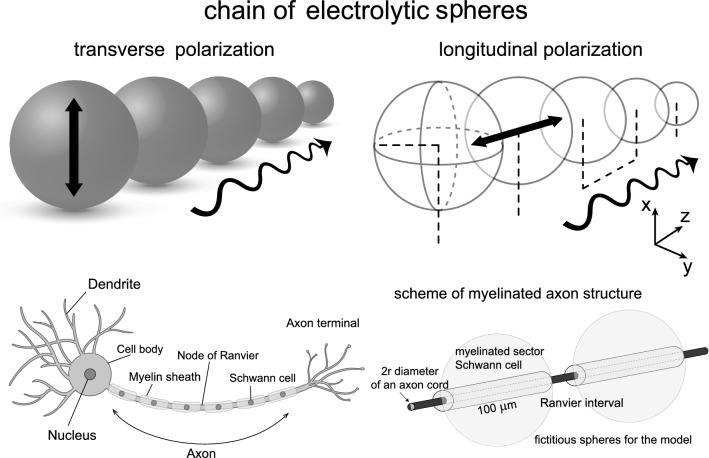
In analogy to metallic nanochains, microchains of finite electrolytic segments ranged with dielectric membranes can be considered; in these electrolytic microchains, the ionic plasmon-polariton modes can propagate similar to in metallic nanochains; we propose to model a periodically myelinated axon as a chain of electrolyte segments, because the plasmon-polaritons can traverse equally discrete and periodically corrugated continuous linear plasmonic system alignments

We propose to apply the model of dipole plasmon-polariton excitations in a linear chain of electrolyte spheres to an axon cord periodically wrapped with myelin sheaths, as schematically depicted in Figs. 1 and 3. The periodicity makes the chain similar to a 1D crystal. Despite the cord of an axon being a continuous ion tube, the modelling of an axon by a chain of segments defined by the periodic myelin sheath meets well with plasmon-polariton kinetics maintaining the same character in discrete chains and in continuous but periodically corrugated wires. The interaction between the chain elements (or segments defined by the periodic myelin sheath) can be regarded as dipole-type coupling. For chains of ionic spheres, the results of the corresponding analysis for metallic chains can be adopted, which supports a dipole model of interaction of chain segments (Citrin 2005; Zhao et al. 2003; Zou et al. 2004).

The dipole interaction resolves itself to the electric and magnetic fields created at any distant point by an oscillating dipole ${\mathbf {D}}({\mathbf {r}},t)$ pinned in ${\mathbf {r}}$, and the electric field dominates this interaction. If the distant point is represented by the vector ${\mathbf {r}}_0$ (with the beginning fixed to the end of ${\mathbf {r}}$, where the dipole is placed), then the electric field produced by the dipole ${\mathbf {D}}({\mathbf {r}},t)$ takes the following form, including the relativistic retardation (Landau and Lifshitz 1973; Jackson 1998),

1 $$\begin{aligned} \begin{array}{ll} &{}{\mathbf {E}} ({\mathbf {r}},\mathbf {r_0},t)=\frac{1}{\varepsilon } \left( -\frac{\partial ^2}{v^2\partial t^2} \frac{1}{r_0} -\frac{\partial }{v \partial t} \frac{1}{r_0^2}-\frac{1}{r_0^3}\right) {\mathbf {D}}({\mathbf {r}},t-r_0/v)\\ &{}\quad + \frac{1}{\varepsilon } \left( \frac{\partial ^2}{v^2\partial t^2}\frac{1}{r_0}+\frac{\partial }{v \partial t} \frac{3}{r_0^2}+\frac{3}{r_0^3}\right) {\mathbf {n}}_0({\mathbf {n}}_0\cdot {\mathbf {D}}({\mathbf {r}}, t-r_0/v)),\\ \end{array} \end{aligned}$$

with ${\mathbf {n}}_0=\frac{{\mathbf {r}}_0}{r_0}$ and $v=\frac{c}{\sqrt{\varepsilon }}$, *c* is the light velocity. The terms with denominators of $r_0^3$, $r_0^2$, and $r_0$ are usually referred to as the near-field, medium-field, and far-field components of the interaction, respectively. The formula above will serve to describe the mutual interaction of the plasmon dipoles at each sphere in the chain. The spheres in the chain are numbered by integers *l*, and the equation for the surface plasmon oscillation of the *l*th sphere can be written as follows (where *d* denotes the separation between the centers of the spheres),

2 $$\begin{aligned} \begin{array}{l} \left[ \frac{\partial ^2}{\partial t^2}+ \frac{2}{\tau _0} \frac{\partial }{\partial t} +\omega _1^2\right] D_{\alpha }(ld,t)\\ \quad =\varepsilon \omega _1^2a^3 \sum \limits _{m=-\infty , \;m\ne l }^{m=\infty } E_{\alpha }\left( md,t-\frac{|l-m|d}{v}\right) \\ \qquad +\varepsilon \omega _1^2a^3 E_{L\alpha }(ld,t) +\varepsilon \omega _1^2a^3 E_{\alpha }(ld,t),\\ \end{array} \end{aligned}$$

$\alpha =z$ indicates the longitudinal polarization, whereas $\alpha =x(y)$ the transverse polarization (the chain orientation is assumed to be along the *z* direction, as illustrated in Fig. 1). The first term on the right-hand side of Eq. (2) describes the dipole coupling between the spheres, and the other two terms correspond to the plasmon attenuation due to Lorentz friction radiation losses and the force field arising from an external electric field, respectively; $\omega _1=\frac{\omega _p}{\sqrt{3\varepsilon }}$ is the self-frequency of the dipole surface plasmons. Ohmic losses are included via the term $\frac{2}{\tau _0}$ similar to what is applied to metals (Brongersma et al. 2000) but with the Fermi velocity of electrons in metals substituted by the mean velocity of ions for a nondegenerated classical Boltzmann distribution regardless of the quantum statistics of ions, i.e.,

3 $$\begin{aligned} \frac{1}{\tau _0}=\frac{{\mathsf {v}}}{2\lambda _\mathrm{B}}+\frac{C {\mathsf {v}}}{2a}, \end{aligned}$$

where $\lambda _\mathrm{B}$ is the mean free path of the carriers (ions) in the bulk electrolyte, ${\mathsf {v}}$ is the mean velocity of the carriers at temperature *T*, ${\mathsf {v}}=\sqrt{\frac{3k_\mathrm{B}T}{m}}$, *m* is the mass of the ion, $k_\mathrm{B}$ is the Boltzmann constant, *C* is a constant on the order of unity (to account for the type of scattering of carriers by the system boundary) and *a* is the radius of a sphere. The first term in the expression for $\frac{1}{\tau _0}$ approximates ion scattering losses such as those occurring in the bulk electrolyte (collisions with other ions, solvent and admixture atoms), whereas the second term describes losses due to the scattering of ions on the boundary of a sphere of radius *a*. According to Eq. (1), we can write the following quantities that appear in Eq. (2),

4 $$\begin{aligned} \begin{array}{ll} E_z(md,t)=&{}\frac{2}{\varepsilon d^3} \left( \frac{1}{|m-l|^3}+\frac{d}{v|m-l|^2}\frac{\partial }{\partial t}\right) \\ &{}\times D_z(md,t-|m-l|d/v),\\ E_{x(y)}(md,t)&{}=-\frac{1}{\varepsilon d^3}\left( \frac{1}{|m-l|^3}+\frac{d}{v|m-l|^2}\frac{\partial }{\partial t}+ \frac{d^2}{v^2 |l-d|}\frac{\partial ^2}{\partial t^2} \right) \\ &{}\times D_{x(y)}(md, t-|m-l|d/v).\\ \end{array} \end{aligned}$$

Because of the periodicity of the chain, a wave-type collective solution of the dynamical equation in the form of Fourier component can be assumed (2),

5 $$\begin{aligned} \begin{array}{l} D_{\alpha }\left( ld,t\right) =D_{\alpha }\left( k,t\right) \mathrm{e}^{-ikld},\\ \quad 0\le k \le \frac{2\pi }{d}. \end{array} \end{aligned}$$

In the Fourier picture of Eq. (2) (the discrete Fourier transform (DFT) with respect to the positions and the continuous Fourier transform (CFT) with respect to time) this solution takes a form similar to that of the solution for phonons in 1D crystals. Note that DFT is defined for a finite set of numbers; therefore, we consider a chain with $2N+1$ spheres, i.e., a chain of finite length $L= 2Nd$. Then, for any discrete characteristic $f(l),\;\;l=-N,\ldots ,0,\ldots ,N$ of the chain, such as a selected polarization of the dipole distribution, we must consider the DFT picture $f(k)=\sum \nolimits _{l=-N}^{N}f(l)\mathrm{e}^{ikld}$, where $k=\frac{2\pi }{2Nd}n,\;n=0,\ldots ,2N$. This means that $kd\in [0,2\pi )$ because of the periodicity of the equidistant chain. The Born–Karman periodic boundary condition, $f(l+L)=f(l)$, is imposed on the entire system, resulting in the form of *k* given above. For a chain of infinite length, we can take the limit $N\rightarrow \infty$, which causes the variable *k* to become quasi-continuous, although $kd\in [0,2\pi )$ still holds.

The Fourier representation of Eq. (2) takes the following form,

6 $$\begin{aligned} \begin{array}{l} \left( -\omega ^2-i\frac{2}{\tau _0}\omega +\omega ^2_1\right) D_{\alpha }(k,\omega )\\ \quad =\omega _1^2\frac{a^3}{d^3}F_{\alpha }(k,\omega )D_{\alpha }(k,\omega )+ \varepsilon a^3 \omega _1^2 E_{0\alpha }(k,\omega ),\\ \end{array} \end{aligned}$$

with

7 $$\begin{aligned} \begin{array}{l} F_z(k,\omega )=4\sum \limits _{m=1}^\infty \left( \frac{\cos (mkd)}{m^3}\cos (m\omega d/v)\right. \\ \quad \left. +\omega d /v \frac{\cos (mkd)}{m^2}\sin (m\omega d/v)\right) \\ \quad +2i \left[ \frac{1}{3}(\omega d /v)^3+2\sum \limits _{m=1}^\infty \left( \frac{\cos (mkd)}{m^3}\sin (m\omega d/v)\right. \right. \\ \quad \left. \left. -\omega d/v\frac{\cos (mkd)}{m^2}\cos (m\omega d/v)\right) \right] ,\\ F_{x(y)}(k,\omega )=-2\sum \limits _{m=1}^\infty \left( \frac{\cos (mkd)}{m^3}\cos (m\omega d/v)\right. \\ \quad \left. +\omega d /v \frac{\cos (mkd)}{m^2}\sin (m\omega d/v) -(\omega d/v)^2\frac{\cos (mkd)}{m}\cos (m\omega d/v)\right) \\ \quad -i \left[ -\frac{2}{3}(\omega d /v)^3+2\sum \limits _{m=1}^\infty \left( \frac{\cos (mkd)}{m^3}\sin (m\omega d/v)\right. \right. \\ \quad \left. \left. +\omega d/v\frac{\cos (mkd)}{m^2}\cos (m\omega d/v) -(\omega d/v)^2\frac{\cos (mkd)}{m}\sin (m\omega d/v)\right) \right] .\\ \end{array} \end{aligned}$$

Similar to metallic nanochains, $\mathrm{Im} F_{\alpha }(k,\omega )\equiv 0$ (for $\alpha =z,x(y)$), which indicates perfect quenching of the radiation losses at any sphere in the chain (meaning that to each sphere, the amount of energy that comes in from the other spheres is the same as the energy outflow due to Lorentz friction). We can easily verify this property, as the related infinite sums in Eq. (7) can be found analytically (Gradshteyn and Ryzhik 1994). Radiation losses occur, however, if the chain with the plasmon-polariton is embedded in another electrolyte, which will be addressed later.

Equation (6) is highly nonlinear with respect to the complex $\omega$ and can be solved both perturbatively in an analytical manner (Jacak 2013) or numerically even beyond the perturbation approach (Jacak 2014). The solutions determined for $\mathrm{Re}\,\omega$ and $\mathrm{Im}\,\omega$ (i.e., for the self-frequency and damping of the plasmon-polaritons, respectively) can be applied to the initial axon model as an effective chain of electrolyte spheres with ion concentrations adjusted to the actual neuron parameters.

**Fig. 2:**
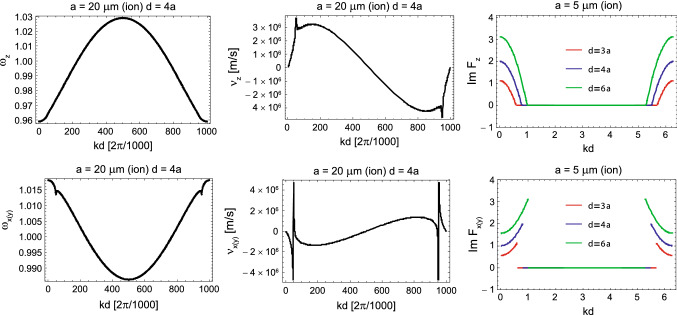
Exact solution for the self-frequencies of the longitudinally and transversely polarized modes of the plasmon-polaritons in an electrolyte chain ($\omega$ in units of $\omega _1$) obtained by solving of Eq. (6) in the region $kd\in [0,2\pi$) (left) and the corresponding group velocities for both types of polarization, $v_g=\frac{\mathrm{d}\omega }{\mathrm{d}k}$ (central); damping of plasmon-polaritons, i.e., the functions $\mathrm{Im}F_{z}(k;\omega =\omega _1)$ and $\mathrm{Im}F_{x(y)}(k;\omega =\omega _1)$ for chains of electrolyte spheres of radius *a* with separations of $d=3a$, 4*a*, and 6*a*, the shift of the singularities toward the band edges with decreasing *d*/*a* is noticeable (right)

The resonance frequency ($\mathrm{Re}\,\omega (k)$), its *k*-derivative, which is the group velocity, and the attenuation rate (in dielectric surroundings) ($\mathrm{Im}\,\omega (k)$) of the dipole plasmon-polariton modes numbered by the wave vector *k*, derived by the solution of Eq. (6) (Jacak 2013, 2014) for ionic chains with example concentrations, chain size and ion parameters, are plotted in Fig. 2.

## Plasmon-polariton model of saltatory conduction: fitting the plasmon-polariton kinetics to the axon parameters

The bulk plasmon frequency is $\omega _p=\sqrt{\frac{q^2 n 4 \pi }{m}}$ (in Gauss units, or $\sqrt{\frac{q^2 n}{\varepsilon _0 m}}$ in SI) (we assumed for the model that the ion charge is $q=1.6 \times 10^{-19}$ C and the ion mass is $m=10^4 m_\mathrm{e}$, where $m_\mathrm{e}=9.1 \times 10^{-31}$ kg is the mass of an electron) and for a concentration of $n=2.1 \times 10^{16}$ 1/m$^3$, we obtain the Mie-type frequency for ionic dipole oscillations, $\omega _1 \simeq \frac{\omega _p}{\sqrt{3 \varepsilon _1}}\simeq 4 \times 10^6$ 1/s, where the relative permittivity of water is $\varepsilon _1 \simeq 80$ for frequencies in the MHz range (Meissner and Wentz 2004) (though for higher frequencies, beginning at approximately 100 GHz, this value decreases to approximately 1.7, corresponding to the optical refractive index of water, $\eta \simeq \sqrt{\varepsilon _1}=1.33$). The axon consists of a cord with a small diameter of 2*r* (for the model we assumed here unrealistically small, $r=3.4$ nm), and this thin cord is wrapped with a myelin sheath of a length of 2*a* per segment; however, for the effective model, we consider fictitious electrolyte spheres of radius *a*. Thus, the auxiliary concentration *n* of ions in the fictitious spheres corresponds to an ion concentration in the cord of $n'=\frac{n 4/3 \pi a^3}{2a \pi r^2}$, which yields a typical concentration of ions in a nerve cell of $n' \sim 10$ mM (i.e., $\sim 6\times 10^{24}$ 1/m$^3$). This is because all the ions participating in the dipole oscillation correspond in the sphere model to a much smaller volume in the real system, that of the thin cord portion (the insulating myelin sheath consists of a lipid substance without any ions). For a model calculation we assumed here smaller than realistic radius of the axon cord, $r=3.4$ nm. This is caused by the fact that for a more realistic larger diameter of the axon cord the plasmon-polariton frequency would be too large, giving too high a speed of the plasmon-polariton signal. The above calculation illustrates here that the MHz frequency of plasmons results in the correct value of the plasmon-polariton speed. Here we have not yet accounted for another mechanism for reducing the plasmon frequency, which is the role of the myelin sheath thickness (tentatively substituted by the reducing of *r*). If this mechanism (as described in the next paragraph) is included the required frequency of plasmon is achieved at the realistic diameter of the axon cord.

The insulating, relatively thick myelin coverage creates a periodically broken channel (corrugated conductor-insulator structure) required for plasmon-polariton formation and its wave-type propagation. To reduce the coupling with the surrounding inter-cellular electrolyte and protect against any leakage of plasmon-polaritons, the myelin sheath must be sufficiently thick, much thicker than what is required merely for electrical insulation.

Here, we considered an initial model of the dielectric surroundings. In the electrolytic surroundings, plasmon oscillations in separated myelinated segments have strongly red shifted self-frequency due to non-radiative damping of plasmons. The dipole oscillating in a segment induces the oppositely oscillating dipole in the surrounding electrolyte, which dissipates energy into heat eventually. This large effect causes the reducing of the $\omega _1$ frequency, according to the damped oscillator scheme, $\omega _1'=\omega _1\sqrt{1-\frac{1}{\tau ^2 \omega _1^2}}$, where $\frac{2}{\tau }$ is the overall damping rate for plasmons in a single chain element. This damping causes a similar increase of damping of the plasmon-polariton (Jacak 2019). Inclusion of this damping and related of the plasmon self-frequency gives the frequency of the order of MHz for a realistic diameter of the axon, $r=2{-}3\,\upmu$m.

**Fig. 3:**
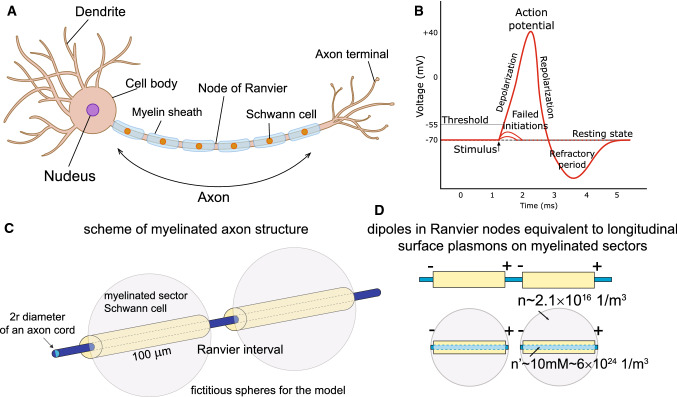
Schematic illustration of a long axon with a chain of periodically repeated myelinated sectors of approximately 100 $\upmu$m in length separated by unmyelinated Ranvier nodes, corresponding to a number of segments of order 10,000 per 1 m of axon length (**a**); time-pattern of the AP forming on a Ranvier node (**b**); periodic fragments of a myelinated axon with a fictitious periodic chain of spherical ionic systems proposed as an effective model (**c**); the equivalence of polarized Ranvier nodes with longitudinal surface plasmons on myelinated sectors (effective concentration of ions *n* in the auxiliary sphere corresponds to the actual ion concentration $n'$) (**d**)

**Fig. 4:**
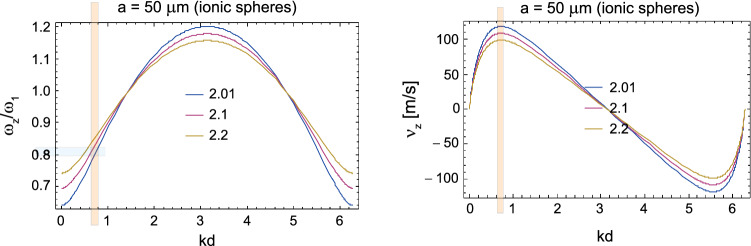
Solutions for the self-frequencies and group velocities of the longitudinal mode of a plasmon-polariton in the model ionic chain; $\omega$ is presented in units of $\omega _1$, here $\omega _1=4 \times 10^6$ 1/s, for a chain of spheres with radius $a=50\,\upmu$m and Ranvier separation *d*, $d/a=2.01$, 2.1, or 2.2, for an equivalent ion concentration in the inner ionic cord of the axon of $n'\sim 10$ mM. Region of *k*, shadowed in the figure-right panel, corresponds to an optimal wave packet with velocity 100 m/s, the corresponding region of frequency is shadowed in the left panel. Any shift in frequency causes lowering of the group velocity

For the resulting plasmon self-frequency in each segment, $\omega _1\simeq 4 \times 10^6$ 1/s, one can determine the plasmon-polariton mode frequencies in a chain of segments of length $2a= 100\,\upmu$m (for a Schwann cell length of 2*a*) and for small chain separations of $d/a=2.01$, 2.1, and 2.2 (corresponding to Ranvier node lengths of 0.5, 5, and $10\,\upmu$m, respectively) within the approach presented above (via the solution of Eq. (6)). The derivative of the obtained self-frequencies with respect to the wave vector *k* determines the group velocities of the plasmon-polariton modes. The results are presented in Fig. 4. We observe that for the ionic system parameters listed above, the group velocity of the plasmon-polaritons easily reaches 100 m/s. The longitudinal mode is polarized suitable to the prolate geometry of segments, assuming that the initial post-synaptic AP or that from the synapse hillock predominantly excites longitudinal ion oscillations. Moreover, what is even more important, the geometry of myelinated segments separated by nodes of Ranvier with the ion inter-membrane channels gated by the voltage prefers the longitudinal modes of plasmon-polaritons propagating along the axon, which are able to polarize/depolarize consecutive nodes of Ranvier (cf. Fig. 3d) in contrast to the transverse polarized modes. The complete theory of plasmon-polariton in a chain of confined electrolyte segments accounts for both longitudinal and transverse polarization modes (Jacak 2015c), but apparently the transverse modes cannot be synchronized with the HH cycles at Ranvier nodes (they cannot polarize/depolarize the nodes of Ranvier), and thus are quickly damped as not supplemented in energy. This circumstance supports also the approximation of dipole-type interaction between surface plasmons oscillating at the chain segments, though in general, especially in a spherical model for chain segments and transverse modes, higher multipole corrections might contribute.

From Fig. 4 we see that it is possible to arrange the wave packet (by selection of appropriate subset of *k* as shadowed in the right panel of the figure) which can propagate with the appropriately high velocity. This subset of *k* determines also the frequency of plasmon-polariton as visualized by shadowing in the left panel in the figure. The mechanism which selects the appropriate *k* range is the HH mechanism at nodes of Ranvier and the thickness of the myelin sheath. The frequency of the plasmon polariton must be adjusted to the characteristic time of the triggering of the opening of Na$^+$ channels at each node of Ranvier (we assume it at the microsecond level). MHz frequency allows for synchronization with $\upmu$s time of gate triggering. Such a mode of plasmon-polariton is stabilized in contrast to modes with larger frequencies. For larger frequency the *k* region right-shifts, which causes lowering of the velocity of plasmon-polariton—cf. Fig. 4, whereas for lower frequency the *k* region left-shifts, which also causes lowering of the velocity on the left side of the maximum.

The regulatory role is played here by the thickness of the myelin layer (as detailed in the next section). The dipole oscillating in a myelinated segment of the axon cord induces in the outer intercellular electrolyte the opposite dipole. Both dipoles coupled across the myelin layer of thickness $\xi$ create the pair of coupled oscillators with beating frequency determined by $\xi$. This beating frequency can be precisely tuned by $\xi$ to a required value at which the frequency of the plasmon-polariton is synchronized with the gating-time of Na$^+$ channels, and thus synchronized with HH cycle at nodes of Ranvier. Such a selected mode of plasmon-polariton is continuously supplied with energy form the AP formation at Ranvier nodes. This mode is thus not damped and propagates with the optimal velocity. Other frequency modes are damped as their energy losses are not covered by the AP formation out of synchronization. A thinner myelin sheath causes an increase of the beating frequency of the dipole oscillator pair, which rises the frequency of plasmon-polariton mode but lowers its velocity, as visible from Fig. 4. Too thick a myelin sheath is also inconvenient—it causes lowering of beating frequency, thus lowering the frequency of the plasmon-polariton mode and with also reduced velocity (the left side with respect to maximum velocity in Fig. 4). Such behavior agrees with observations of the saltatory conduction velocity lowering at demyelination syndromes like Multiple Sclerosis.

Note that for $\omega _1=4\times 10^6$ 1/s and $a=50 \,\upmu$m, the light-cone interference conditions $kd-\omega _1 d/c=0$ and $kd+\omega _1 d/c=2\pi$ are fulfilled for extremely small values of *kd* and $2\pi -kd$, respectively, (of the order of $10^{-6}$ for $d/a\in [2,2.5]$) and thus are negligible with regard to the plasmon-polariton kinetics [though are important for metals (Jacak et al. 2015; Jacak 2014)]. The related singularities on the light-cone induced by the far- and medium-field contributions to the dipole interaction are pushed to the borders of the *k* domain and thus are unimportant for the considered ionic system. Hence, the quenching of the radiative losses (i.e., the perfect balance the Lorentz friction in each segment by the radiation income from the other segments in the chain) for the plasmon-polariton modes in the axon model occurs practically throughout the entire $kd\in [0,2\pi )$ region. Additionally, the aforementioned singularities (Jacak 2014) are characteristic of infinite chains and therefore cannot fully develop because the nerve model electrolyte chains are of a finite length, whereas other effects, such as quenching of irradiation losses, occur for finite chains due to very fast convergence of sums in Eq. (7) with denominators $m^2$ and $m^3$ (practically, a chain consisting of only 10 segments exhibits almost the same properties as an infinite chain).

**Fig. 5:**
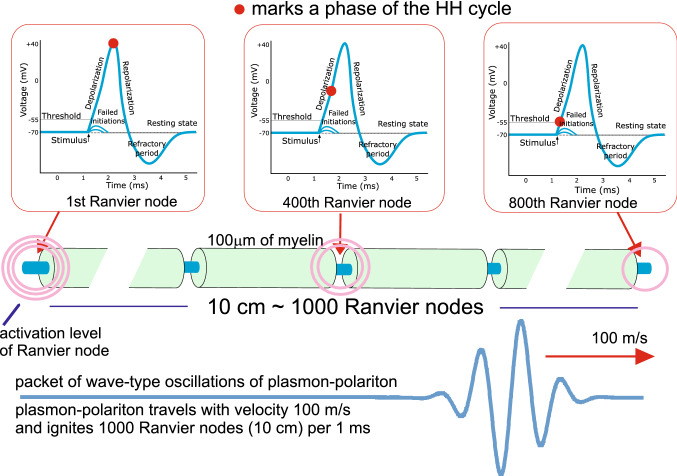
Schematic presentation of the firing of 10 cm long myelinated axon. The wave-packet of plasmon-polariton oscillations travels along the axon with the velocity of 100 m/s and in time of 1 ms ignites 1000 Ranvier nodes (as the myelinated segments have a length of 100 $\upmu$m each). The Hodgkin–Huxley (HH) cycle of the AP spike creation takes ca. 1 ms, thus consecutive Ranvier nodes are in various phases of HH cycle, as indicated by red points. Hence, the whole 10 cm long axon is in firing within the time period of 1 ms

Although the ionic system chain model for a myelinated axon appears to be a crude approximation of the real axon structure, it can serve for the comparison of the energy and time scales of plasmon-polariton propagation implied by the model with the observed kinetic parameters of nerve signals. In the model, the propagation of a plasmon-polariton through the axon chain, excited by an initial AP on the first Ranvier node (after the synapse or, for the reverse signal direction, in the neuron cell hillock), sequentially ignites the consecutive Ranvier node blocks of Na$^+$ and K$^+$ ion gates. The resulting firing of the neuron traverses the axon with a velocity of approximately 100 m/s, consistent with the velocity actually observed in myelinated axons [and not possible for ionic diffusive current with the cable model (Debanne et al. 2011; Brzychczy and Poznański 2011)]. The plasmon-polariton ignition of consecutive Ranvier nodes releases the creation of the same AP pattern aided by the external energy supply at each Ranvier node block by ATPase. Because of the nonlinearity of the HH ion-channel block mechanism, the signal growth saturates at a constant level, and the overall timing of each AP spike has the stable shape of a local polarization/depolarization scheme. The permanent supply of energy associated with creation of the AP spikes at sequentially firing nodes of Ranvier contributes to the plasmon-polariton assuring that its amplitude is beyond the activation threshold. The external energy supply (through the conventional ATP/ADP cell mechanism) assisting HH cycle at each node of Ranvier residually compensates all Ohmic losses of the plasmon-polariton mode propagating along the axon and ensures the undamped propagation over an unlimited range. Although the entire signal cycle of the AP on a single Ranvier node block requires several milliseconds (or even longer when one includes the time required to restore steady-state conditions, which, on the other hand, conveniently blocks the reversing of the signal), subsequent nodes are ignited more rapidly, corresponding to the velocity of the plasmon-polariton wave-packet triggering the ignition of consecutive Ranvier nodes (in a period of one millisecond ca. 1000 nodes of Ranvier are ignited). Thus, we deal with the firing of the axon, which propagates with the velocity of the ionic plasmon-polariton wave-packet of ca 100 m/s (Fig. 5). The direction of the velocity of the plasmon-polariton wave-packet is adjusted to the semi-infinite geometry of the chain (in fact the chain is finite and is excited at one of its ends). The firing of the AP triggered by the plasmon-polariton traverses along the axon in only one direction, because the nodes that have already fired have had their Na$^+$, K$^+$ gates discharged and require a relatively long time to restore their original status (they require a time of the order of even one second and sufficient energy supply to bring the ion concentrations to their steady values via cross-membrane active ion pumps against the concentration gradient).

The plasmon-polariton scheme described above for the ignition of AP spike formation in the ordered chain of Ranvier nodes along an axon is thus consistent with the saltatory conduction observed in myelinated axons. The observations that firing of the AP can simultaneously move in two opposite directions if a certain central node of Ranvier of a passive axon is ignited, as well as the observation of the maintenance of the firing traverse despite small breaks in the axon cord or a few damaged Ranvier nodes also agree with the collective wave-type plasmon-polariton model of saltatory conduction in contrast to the lack of satisfactory explanations in models based on the cable theory. The maintenance of the plasmon-polariton kinetics despite discontinuities in the axon cord agrees well with the discrete chain model.

**Fig. 6:**
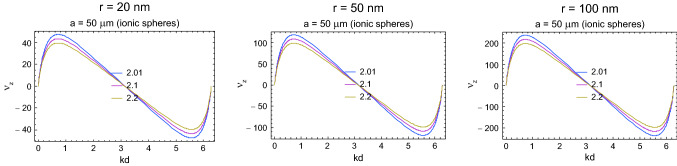
Comparison of the group velocities, in units of m/s, of the longitudinal plasmon-polariton mode with respect to the wave vector $k\in [0,2\pi /d)$ within the axon model for a Schwann cell myelinated sectors with a length of 100 $\upmu$m, Ranvier separations of 0.5, 5, and 10 $\upmu$m (represented by $d/a=2.01$, 2.1, and 2.2 in the figure, respectively) and for the axon cord radii of $r=20$, 50, and 100 nm

In Fig. 6, the group velocity of the plasmon-polariton traversing a firing myelinated axon is plotted for various diameters of the axon internal cord, with a length of $100\,\upmu$m for each myelinated segment wrapped by Schwann cells and Ranvier intervals of 0.5, 5, and 10 $\upmu$m. The dependence of the group velocity on the length of the Ranvier interval is weak (i.e., negligible at the scale considered, which is consistent with the equivalence of the discrete model for the continuous system if one considers wave type plasmon-polariton propagation), but the increase in the velocity with increasing internal cord thickness is significant, similarly to linear increase in real axons with increasing diameters.

To comment on the appropriateness of the chain model for axons, let us note that even though the thin core of the axon is a continuous ion conducting fiber, the surface electromagnetic field can be closely pinned to the linear conductor similarly as to the Goubau line (well-known from microwave technology) (Goubau 1950; Sommerfeld 1899) and if periodically wrapped by dielectric shells, plasmon-polaritons propagate similarly as in a chain. For plasmon-polariton kinetics, the continuity or discontinuity of the conducting fiber is unimportant because we deal here with traversing wave packet of the synchronized plasma oscillations and not of a net current, similarly to Goubau microwave lines, which also have discontinuous segments impossible to be crossed by any current. The Goubau lines maintain their transmittance via discrete disconnected elements. Instead of a chain of spheres one can consider prolate spheroid or elongated cylindrical rod chains. This modification resolves itself to the substitution of the isotropic $\omega _1$ frequency in Eq. (2) with frequencies that are different for each polarization, $\omega _{\alpha 1}$, i.e., $\left[ \frac{\partial ^2}{\partial t^2}+ \frac{2}{\tau _{\alpha 0}} \frac{\partial }{\partial t} +\omega _{\alpha 1}^2\right] D_{\alpha }(ld,t) =\mathcal{{A}} \sum \nolimits _{m=-\infty , \;m\ne l }^{m=\infty } E_{\alpha }\left( md,t-\frac{|l-m|d}{v}\right) +\mathcal{{A}} E_{L\alpha }(ld,t) +\mathcal{{A}} E_{\alpha }(ld,t)$, where $\mathcal{{A}}= V \frac{n q^2}{m}$ is a shape independent factor proportional to the number of ions at concentration *n* in the volume of the spheroid with semiaxes *a*, *b*, *c*, $V=\frac{4\pi }{3}abc = \frac{4 \pi }{3}a^3$ (the latter for a sphere). Taking into account that the plasmon frequency in a bulk electrolyte with ion concentration *n* equals to, $\omega _p=\sqrt{\frac{n q^2 4 \pi }{m}}$, one can rewrite $\mathcal{{A}}$ as follows, $\mathcal{{A}}= \frac{abc \omega _p^2}{3}= \varepsilon a^3 \omega _1^2$ (the latter for a sphere, for which $\omega _1=\frac{\omega _p}{\sqrt{3\varepsilon }}$). The Ohmic losses can be included via the anisotropic term, $\frac{1}{\tau _{\alpha 0}}=\frac{{\mathsf {v}}}{2\lambda _\mathrm{B}}+\frac{C {\mathsf {v}}}{2a^{\alpha }},$ where $a^{\alpha }$ is the dimension (semiaxis) of the spheroid in the direction $\alpha$ (equal to *a*, *b*, *c* for a spheroid). The first isotropic term in the expression for $\frac{1}{\tau _{\alpha 0}}$ approximates ion scattering losses such as those occurring in the bulk electrolyte (thus is isotropic), whereas the second term describes the losses due to scattering of ions on the anisotropic boundary of the spheroid. This term can be neglected for longitudinal polarization because the neuron cord is continuous along the *z* direction. The dipole coupling is independent of the shape of the chain elements. The mutual independence of dipole oscillations with distinct polarization described above follows from the linearity of the dynamics equation (versus the dipole) regardless of the metal or electrolyte conducting elements.

Because the dynamics equation is not affected by the anisotropy, the solutions of the equation for each polarization have the same form as that for the spherical case with the exception of the modification of the related frequency of the dipole oscillations in each direction and the small correction of the orientation dependent contribution of the scattering ratio (this part is related to the boundary scattering of carriers and is not important for longitudinal polarization when the axon cord is continuous). Thus, we can independently renormalize the equation for dipole oscillations for each polarization direction, introducing the oscillation self-frequency for each direction $\omega _{\alpha 1}$ in a phenomenological manner (these frequencies can be estimated numerically, whereas for a sphere, $\omega _1=\frac{\omega _p}{\sqrt{3\varepsilon }}$; in general, the longer semiaxis, the lower the related dipole oscillation frequency is).

The periodic structure of a myelinated axon does not form a chain of electrolyte spheres but rather is a thin electrolyte cord with periodically distributed myelinated sectors separated by very short unmyelinated intervals of Ranvier nodes. The periodic corrugated structure of the dielectric isolation allows, however, for collective plasmon wave-type oscillations, $\sim \mathrm{e}^{iqz}$, with *q* governed by the periodicity. The wave-type propagation has the form of synchronic dipole oscillations of myelinated sectors. These dipole oscillations are equivalent to periodic polarization of Ranvier nodes. The model allows for quantitative estimation of the relevant propagation characteristics and verification of whether plasmon-polariton dynamics fits the observed features of the saltatory conduction in myelinated axons.

The polarization of Ranvier nodes induced by plasmon-polaritons initiates opening of Na$^+$ across-membrane ion channels at nodes of Ranvier, which results in a characteristically large AP signal formation due to the transfer of ions through the open gates caused by the difference in ion concentrations on opposite sides of the membrane. The entire HH cycle at a single node of Ranvier requires several milliseconds, but the initial increase in polarization needed for the rapid opening of the Na$^+$ channel occurs on the microsecond timescale and must be synchronized with the plasmon-polariton frequency.

Plasmon-polaritons do not interact with external electromagnetic waves or, equivalently, with photons (even at adjusted frequency), which is a consequence of the large difference between the group velocity of plasmon-polaritons and the velocity of photons ($c/\sqrt{\varepsilon }$). The resulting large discrepancy between the wavelengths (and momentum) of photons and plasmon-polaritons of the same energy prohibits mutual transformation of these two types of excitations because of momentum-energy conservation constraints. Therefore, plasmon-polariton signaling by means of collective wave-type dipole plasmon oscillations along a chain, i.e., plasmon-polaritons, can be neither detected nor perturbed by external electromagnetic radiation. This also fits well with neuron signaling properties in the PNS and in the white myelinated matter in the CNS. The temperature influences the mean velocity of ions, ${\mathsf {v}}=\sqrt{\frac{3kT}{m}}$, thereby enhancing the Ohmic losses with increasing temperature (cf. Eq. (3)), which in turn strengthens plasmon-polariton damping. Hence, at higher temperatures, higher external energy supplementation is required to maintain the same long-range propagation of plasmon-polaritons with a constant amplitude. This property is also consistent with experimental observations.

## The role of the thickness of the myelin sheath

**Fig. 7:**
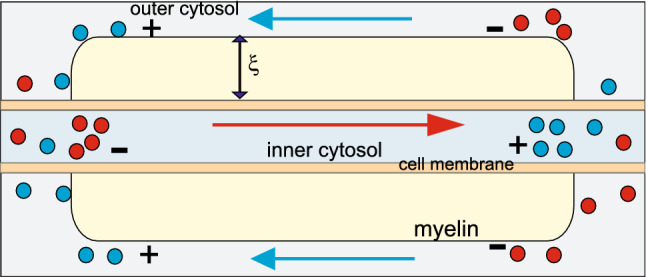
Cartoon of a single myelinated segment—polarization of longitudinal dipole type of the inner cytoplasm inside the axon cell (red arrow) induces local opposite polarization of the outer cytoplasm (blue arrows). Both dipoles oscillate as the pair of coupled oscillators across the insulating myelin layer. The coupling is weak for a sufficiently thick myelin sheath and causes slow beats due to oscillator coupling. At the nodes of Ranvier the coupling across much thinner cell membrane is strong and causes quick beating out of a resonance with ion gate timing, thus damped. Slow beating oscillations of coupled dipoles are ranged only to the myelinated sector

It is known that the thickness of the myelin layer is an essential factor deciding on the proper functioning of myelinated axons. This thickness is greater than that needed for simple isolation because the role of myelin is more specific and not related with electrical isolation. In the case of a plasmon dipole oscillating in the myelinated segment, as part of the plasmon-polariton, the ion oscillations in the axon cord excite the oppositely directed also oscillating dipole of ions in the outer electrolyte, in the cave surrounding the myelinated segment, cf. Fig. 7. Coupling of both dipoles across the myelin sheath of thickness, $\xi$, is of the near-field coupling form. By $d_1(t)$ let us denote the longitudinal dipole in the axon rod in this myelinated segment, and by $d_2$ the dipole in the outer cytosol cave adjacent to the myelinated segment and induced by the $d_1$ dipole. The oppositely directed $d_2$ dipole is activated by $d_1$ and vice versa. The equation for the dynamics of this sub-system is as follows,

8 $$\begin{aligned} \begin{array}{l} \frac{\mathrm{d}^2 d_1}{\mathrm{d} t^2} +\frac{2}{\tau _1} \frac{\mathrm{d} d_1}{\mathrm{d}t} + \omega _1^2 d_1= \mu _1 E_1,\\ \frac{\mathrm{d}^2 d_2}{\mathrm{d} t^2} +\frac{2}{\tau _2} \frac{\mathrm{d} d_2}{\mathrm{d}t} + \omega _2^2 d_2= \mu _1 E_2,\\ \end{array} \end{aligned}$$

where, $\frac{1}{\tau _i}$ is the damping rate of the surface plasmon *i*-th dipole, $\omega _i$ its self-frequency, $\mu _i$ is the longitudinal polarizability of the rod for $i=1$ and of the cave in surrounding cytosol for $i=2$ (the polarizability for a sphere equals to $\frac{a^3 4 \pi n q^2}{3M}$, *a*—the sphere radius, *n*—concentration of ions, *M*—ion mass, *q*—ion charge). The electrical fields induced by dipoles in the near-field zone are, $E_{1(2)}(t)=-\frac{1}{\varepsilon \xi ^3} d_{2(1)}(t-\frac{\xi }{v})$, where $\xi$ is the thickness of the myelin sheath, $v=\frac{c}{\sqrt{\varepsilon }}$, and these fields describe mutual interaction of dipoles $d_1$ and $d_2$, i.e., the electrical induction caused by opposite dipoles in the near-field coupling approximation. Simplifying (for illustration) by the assumption, $\mu _1=\mu _2=\mu$, $\omega _1=\omega _2=\omega _0$, and neglecting here the damping and retardation, Eq. (8) attains the shape,

9 $$\begin{aligned} \begin{array}{l} \frac{\mathrm{d}^2 d_1}{\mathrm{d} t^2} + \omega _0^2 d_1= -\frac{\mu }{\varepsilon \xi ^3} d_2 ,\\ \frac{\mathrm{d}^2 d_2}{\mathrm{d} t^2} + \omega _0^2 d_2= -\frac{\mu }{\varepsilon \xi ^3} d_1 .\\ \end{array} \end{aligned}$$

This is the equation for two coupled harmonic oscillators. It has the self-frequencies, for assumed solution, $d_1(t)=A\mathrm{e}^{i\Omega t+\phi }$ and $d_2(t)=B\mathrm{e}^{i\Omega t +\psi }$, given by,

10 $$\begin{aligned} \mathrm{det}\left[ \begin{array}{ll} -\Omega ^2+\omega _1^2, &\frac{ \mu }{\varepsilon \xi ^3}\\ \frac{\mu }{\varepsilon \xi ^3},&-\Omega ^2+\omega _1^2\\ \end{array} \right] =0, \end{aligned}$$

i.e., $$\Omega _1^2=\omega _0^2+\frac{\mu }{\varepsilon \xi ^3}$$, $$\Omega _2^2=\omega _0^2-\frac{\mu }{\varepsilon \xi ^3}$$.

For the initial condition suitable for excitation of the considered segment of the axon, i.e., $d_1(0)=D$, $d_2(0)=0$, $\frac{\mathrm{d} d_{1(2)}}{\mathrm{d}t }(0)=0$, one gets the beating with low frequency, $\Omega _1-\Omega _2\simeq \frac{\mu }{\varepsilon \xi ^3 \omega _0}$. For sufficiently large $\xi$ we get thus slow oscillations required for the time scale of opening of the Na$^+$ ion channels at nodes of Ranvier to trigger the HH cycle. The period of the igniting signal (with an amplitude beyond the threshold for Na$^+$ ion channel opening) cannot be lower than the characteristic time of the activation of these ion channels.

The frequency for a longitudinal plasmon is reduced in a strongly elongated axon rod segment with the aspect ratio $\sim 10^{-3}$ and additionally reduced by an appropriate increase of $\xi$ due to the beating effect and finally achieves the value of $\sim 10^6$ 1/s, resulting in the saltatory conduction velocity $\sim 100$ m/s. Thus we see, that the myelin layer thickness controls the velocity of the saltatory conduction and simultaneously accommodates the frequency of the igniting signal oscillation (of plasmon-polariton wave packet) to the time scale of triggering of the ion channels at nodes of Ranvier (being of the order of a microsecond). Only this frequency is selected and the corresponding plasmon-polariton wave packet is strengthened by synchronized HH cycles in contrary to other non-synchronized frequency modes of the plasmon-polariton. Non-synchronized modes are quickly damped due to Ohmic losses.

At the Ranvier node, the inner and outer cytosol are separated by the thin bare cell membrane, thus dipole coupling across the thinner barrier is stronger and the corresponding beating quicker. The quick component of this beating is also quenched as not synchronized with the time scale of electrically gated Na$^+$ channels, in contrast to the slow component of the *trans*-myelin beating. Via synchronization with HH cycle ignition time scale, this selected mode of plasmon-polariton is continuously supplemented in energy by HH cycles and simultaneously is able to ignite the HH cycle on consecutive Ranvier nodes along the chain of myelinated segments on arbitrary large distances. This explains why only the myelinated segments oscillate and the frequency of the related plasmon-polariton wave packet is precisely tuned by the myelin thickness, $\xi$. The oscillation of the longitudinal surface plasmon on the myelinated segment of the axon rod is equivalent to the opposite dipole oscillation (polarization) of the node of Ranvier (but it is not its own self-oscillation). This is schematically shown in Fig. 3d.

**Fig. 8:**
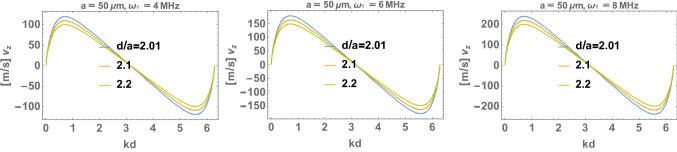
Dependence of the velocity of plasmon polariton longitudinal modes, $v_z$, with respect to the self-frequency of surface plasmon oscillations of the single myelinated segment, $\omega _1$. Strong (almost linear) dependence is visible

**Fig. 9:**
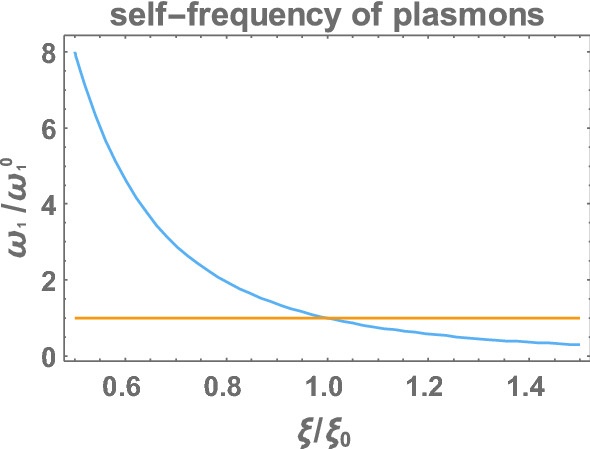
Dependence of the self-frequency of plasmon oscillations in a single myelinated segment, $\omega _1$, versus thickness of the myelin sheath $\xi$, where $\omega _1^0=4 \times 10^6$ Hz corresponds to the thickness $\xi _0$ (at which $v_z=100$ m/s). A sharp increase of the oscillation frequency is visible with diminishing of the thickness $\xi$ (the diminishing by only 20% enhances the frequency twice, by 50% thickness reduction causes eightfold increase of frequency)

The described mechanism of selection of the low self-frequency of plasmon oscillations of the myelinated fragment allows for accommodation of the frequency of plasmon-polariton (via the equation defining dynamics of plasmon-polariton in the chain of myelinated segments, i.e., Eq. (6)). Not all frequency modes of plasmon-polariton are persistent. Ohmic damping causes their attenuation on a short distance unless the energy is supplied by AP formation at consecutive nodes of Ranvier. This requires, however, a perfect coincidence of the plasmon-polariton mode frequency with timing of opening of Na$^+$ ion channels across the axon membrane at nodes of Ranvier. Too quick oscillations are not able to trigger the opening of the gates which eliminates corresponding modes of plasmon-polariton. Too low oscillations reduce velocity of corresponding nodes and are retarded with respect to the optimal ones. This simple mechanism selects the optimal and persistent wave packet of plasmon-polariton with the velocity observed in the saltatory conduction. This wave packet is supplied with energy by AP formation at consecutive nodes of Ranvier. One can state that the thickness of the myelin sheath is a control factor for synchronization of plasmon polariton with the AP formation mechanism needed for overcoming of the Ohmic attenuation of the plasmon-polariton ignition signal over arbitrary large distances. Reducing of the myelin sheath thickness increases frequency of the plasmon-polariton which causes, however, de-synchronization with opening time of Na$^+$ channels, which perturbs plasmon-polariton kinetics, just like in Multiple Sclerosis (Figs. 8, 9).

## Micro-saltatory conduction in C fibers

Recently, it has been discovered that in the thin (ca. 0.1 $\upmu$m for diameter) unmyelinated axons in the PNS intermembrane Na$^+$ channels cluster in a lipid rafts periodically distributed along axons. This localized concentration of Na$^+$ channels resembles in structure the ion channel organization at the nodes of Ranvier in thicker myelinated axons, yet it is currently unknown whether this translates into an equivalent phenomenon of saltatory conduction or related-functional benefits and efficiencies. However, observations indicate that AP signal transduction velocity in these, so-called C fibres, at least 10 times exceeds the theoretical cable model estimations (Neishabouri and Faisal 2014). C fibers are very thin and long unmyelinated peripheral axons responsible for transmitting nociceptive pain sensations (cf. references in Neishabouri and Faisal 2014). The clustering of Na$^+$ channels on lipid rafts resembles the structure of nodes of Ranvier in myelinated fibers are typically of length 0.1–0.3 $\upmu$m and span 5–10 $\upmu$m. They are suggested to permit micro-saltatory conduction in those thin axons despite they are unmyelinated (Neishabouri and Faisal 2014). This emphasizes that the central role for the saltatory conduction plays the periodicity of some structure imposed on a net axon. The periodicity allows the wave type propagation of a triggering signal in the form of ion plasmon-polariton which ignites the consecutive blocks of Na$^+$ channels where the formation of AP takes place according to HH mechanism (K$^+$ channels are still randomly distributed). The synchronized ion oscillations in periodic internodal segments (between lipid rafts along the C fiber) can be organized as well without any myelin sheath. The myelin plays only the regulatory role as described above to precisely accommodate the plasmon-polariton mode to Na$^+$ gates. In both cases, of myelinated and unmyelinated periodic structures, the plasmon-polariton signal is ca. 1–2 orders of magnitude quicker than the ordinary diffusion current of ions along an axon (Neishabouri and Faisal 2014).

The AP formation at lipid rafts (similarly as at nodes of Ranvier) is of major importance for the selection of an appropriate mode of plasmon-polariton—that one which is synchronized with HH cycles at consecutive nodes. Only such a mode will be persistent due to energy supplementation at HH cycles. Other, non-synchronized modes are quickly quenched due to strong damping of plasmon-polaritons on short distances. The plasmon-polariton is not a local effect in contrast to the diffusion current described by cable theory. The plasmon-polariton is disjoint with the HH mechanism at nodes but triggers the HH cycles via depolarization of Na$^+$ gates beyond the required threshold. For this triggering the frequency of plasmon-polariton cannot be larger than the inverse time of the activation of ion gates. This condition is satisfied by MHz frequency, thus higher frequency modes cannot be synchronized with HH cycles and without energy supplementation are eliminated abolishing effective communication. Lower frequencies are also to be synchronized with ion gates but for them the group velocity of plasmon polarization mode is smaller, so are retarded in comparison to the quickest one and also do not ignite HH cycles at consecutive nodes, because these cycles are already ignited by the quickest synchronized mode.

This scenario explains the micro-saltatory conduction in C fibers. The absence of the myelin sheath causes the frequency of the plasmon-polariton in C fibers to be larger than in myelinated axons and mitigated only by the strong damping of surface plasmon oscillations in periodic segments. This damping is caused by the large Ohmic losses in the inner neuron cytosol and by a strong coupling to outer electrolyte across the membrane. The latter is especially large in the absence of myelin as in C fibers. Thus for these fibers we get the self-frequency for plasmons at a single segment, $\omega _1'=\omega _1\sqrt{1-\frac{1}{\tau ^2\omega _1^2}}$ (where $\frac{2}{\tau }$ represent the damping rate), strongly reduced but still larger than required for the synchronization with ion gate triggering time. Hence, the mode of plasmon-polariton will be selected with as much as possible a lowered frequency of plasmon polariton related to $\omega _1'$ as in Fig. 4 (left panel, left low corner). This does not give an extreme velocity to the saltatory conduction according to Fig. 4 (right panel), though highly exceeding the diffusion of ions. Only the inclusion of the myelin reduces transmembrane energy losses and, on the other hand, gives the mechanism of lowering of plasmon-polariton frequency precisely controlled by the myelin thickness. In the latter case the maximal velocity mode (the maximal in Fig. 4 (right panel)) can be synchronized with ion gates and HH cycles. Therefore in the case of C fibers the saltatory conduction does not reach as high speed as in the myelinated axons but still at least 10 times larger than the diffusion current. The diffusion current coupled locally with HH cycles (as in the conventional models, cf. Neishabouri and Faisal 2014) is simply too slow and highly retarded, thus even when excited does not play any communication role here in the already fired axon.

It is thus evident that the cable theory (coupled with HH cycle of AP formation) is helpful only in small short dendrites and unmyelinated axons without any periodic structure of ion gate clusters. Periodicity (by the myelination separated by nodes of Ranvier or by periodic rafts with Na$^+$ gate clusters) always causes plasmon-polariton kinetics which if synchronized with HH cycles is much more efficient and energy efficient, persistent and quick over long distances. We see then no problem in the high velocity of a plasmon-polariton signal which can be very large for high frequency modes. The problem is that such high frequency modes are outside the synchronization with ion gates and are quickly eliminated as strongly damped and thus useless. Only synchronized with Na$^+$ gates can the plasmon-polariton mode propagate on long distances despite strong damping because of the energy supplementation at each node with an HH cycle.

## Conclusion

The utilization of the radiatively undamped plasmon-polariton propagation in a chain of electrolyte subsystems may explain efficient and long range saltatory conduction in myelinated axons in the peripheral neural system and in the white matter of the brain and spinal cord. The effective plasmon-polariton model of the triggering of AP firing along an axon myelinated by Schwann cells separated by nodes of Ranvier fits well with the high conduction velocity observed at the saltatory conduction in agreement with the temperature and size dependence (with respect to the diameter of the axon) of the conduction velocity. This coincidence together with the immunity of plasmon-polaritons to external e-m perturbations or detection support the reliability of the new model proposed for the saltatory conduction in myelinated axons, which is very efficient, quick and energy efficient despite the poor ordinary conductivity of axons.

Plasmon-polariton kinetics of the triggering signal along the myelinated axons has the observed velocity and exceeds by 1–2 orders any estimations based on the conventional cable theory. The role of myelin thickness is elucidated as the regulatory factor in a different manner than for diffusion of ions. The plasmon-polariton is a nonlocal effect decoupled from HH cycles at Ranvier nodes, though the latter select the appropriate mode of plasmon polariton via synchronization with triggering of Na$^+$ channels.

A plasmon-polariton model of the saltatory conduction explains also the recently observed micro-saltatory conduction in unmyelinated ultra thin C fibers in the PNS which possess the periodic structure of rafts with clusters of Na$^+$ gates along the net axon. In these long axons important for pain sensation the velocity of signal triggering the axon firing is lower than in myelinated axons but still by at least one order of magnitude exceeds the diffusion velocity in this case.

It is worth noting some quantum aspects of plasmon-polaritons, which are synchronized in a wave form simultaneous for plasmon oscillations in all segments of a chain. The plasmon oscillations in a particular segment are, however, of quantum nature (regardless of whether electrons or ions), though seem to be quite ordinary oscillations of charge fluctuations. Plasmons are coherent oscillations of all charges in the subsystem and this coherence can be understood exclusively in quantum terms (e.g., of random phase approximation by Pines, Bohm Pines 1999).

## Electronic supplementary material

Below is the link to the electronic supplementary material.

Supplementary Material

## References

[CR1] Barnes WL, Dereux A, Ebbesen TW (2003). Surface plasmon subwavelength optics. Nature.

[CR2] Berini P (2009). Long-range surface plasmon polaritons. Adv Opt Photonics.

[CR3] Brongersma ML, Hartman JW, Atwater HA (2000). Electromagnetic energy transfer and switching in nanoparticle chain arrays below the diffraction limit. Phys Rev B.

[CR4] Brzychczy S, Poznański R (2011). Mathematical neuroscience.

[CR5] Citrin D (2004). Coherent excitation transport in metal-nanoparticle chains. Nano Lett.

[CR6] Citrin DS (2005). Plasmon polaritons in finite-length metal-nanoparticle chains: the role of chain length unravelled. Nano Lett.

[CR7] Citrin D (2006). Plasmon-polariton transport in metal-nanoparticle chains embedded in a gain medium. Opt Lett.

[CR8] Cooley J, Dodge F (1966). Digital computer solutions for excitation and propagation of the nerve impulse. Biophys J.

[CR9] Dayan P, Abbott LF (2001). Theoretical neuroscience: computational and mathematical modeling of neural systems.

[CR10] de Abajo FJG (2010). Optical excitations in electron microscopy. Rev Mod Phys.

[CR11] Debanne D, Campanac E, Białowa̧s A, Carlier E, Alcaraz G (2011). Axon physiology. Physiol Rev.

[CR12] Ermentrout GB, Terman DH (2010). Mathematical foundations of neuroscience. Interdisciplinary applied mathematics.

[CR13] FitzHugh R (1961). Impulses and physiological states in theoretical models of nerve membrane. Biophys J.

[CR14] FitzHugh R (1973). Dimensional analysis of nerve models. J Theor Biol.

[CR15] Forrest MD (2014). Can the thermodynamic Hodgkin–Huxley model of voltage-dependent conductance extrapolate for temperature?. Computation.

[CR16] Fribance S, Wang J, Roppolo JR, de Groat WC, Tai C (2016). Axonal model for temperature stimulation. J Comput Neurosci.

[CR17] Goldman L, Albus J (1968). Computation of impulse conduction in myelinated fibers; theoretical basis of the velocity-diameter relation. Biophys J.

[CR18] Goubau G (1950). Surface waves and their application to transmission lines. J Appl Phys.

[CR19] Gradshteyn IS, Ryzhik IM (1994). Table of integrals series and products.

[CR20] Hodgkin A, Huxley A (1952). A quantitative description of membrane current and its application to conduction and excitation in nerve. J Physiol (Lond).

[CR21] Huidobro PA, Nesterov ML, Martin-Moreno L, Garcia-Vidal FJ (2010). Transformation optics for plasmonics. Nano Lett.

[CR22] Izhikevich EM (2007). Dynamical systems in neuroscience: the geometry of excitability and bursting.

[CR23] Jacak WA (2013). On plasmon polariton propagation along metallic nano-chain. Plasmonics.

[CR24] Jacak W (2014). Exact solution for velocity of plasmon-polariton in metallic nano-chain. Opt Express.

[CR25] Jacak WA (2015). Size-dependence of the Lorentz friction for surface plasmons in metallic nanospheres. Opt Express.

[CR26] Jacak W (2015). Plasmons in finite spherical electrolyte systems: RPA effective jellium model for ionic plasma excitations. Plasmonics.

[CR27] Jacak W (2015). Propagation of collective surface plasmons in linear periodic ionic structures: plasmon polariton mechanism of saltatory conduction in axons. J Phys Chem C.

[CR28] Jacak W (2019). Nonradiative energy losses of plasmon-polariton in a metallic nano-chain deposited on a semiconductor substrate. Plasmonics.

[CR29] Jacak J, Krasnyj J, Jacak W, Gonczarek R, Chepok A, Jacak L (2010). Surface and volume plasmons in metallic nanospheres in semiclassical RPA-type approach; near-field coupling of surface plasmons with semiconductor substrate. Phys Rev B.

[CR30] Jacak W, Krasnyj J, Chepok A (2015). Plasmon-polariton properties in metallic nanosphere chains. Materials.

[CR31] Jack J, Noble D, Tsien R (1983). Electric current flow in excitable cells.

[CR32] Jackson JD (1998). Classical electrodynamics.

[CR33] Keener J, Sneyd J (2009). Mathematical physiology.

[CR34] Landau LD, Lifshitz EM (1973). Field theory.

[CR35] Lazarevich IA, Kazantsev VB (2013). Dendritic signal transmission induced by intracellular charge inhomogeneities. Phys Rev E.

[CR36] Maier SA (2007). Plasmonics: fundamentals and applications.

[CR37] Maier SA, Atwater HA (2005). Plasmonics: localization and guiding of electromagnetic energy in metal/dielectric structures. J Appl Phys.

[CR38] Markel VA, Sarychev AK (2007). Propagation of surface plasmons in ordered and disordered chains of metal nanospheres. Phys Rev B.

[CR39] Meissner T, Wentz F (2004). The complex dielectric constant of pure and sea water from microwave satellite observations. IEEE THRS.

[CR40] Mie G (1908). Beiträge zur optik trüber medien, speziell kolloidaler metallösungen. Annalen der physik.

[CR41] Moore JW, Joyner RW, Brill MH, Waxman SD, Najar-Joa M (1978). Simulations of conduction in uniform myelinated fibers. Relative sensitivity to changes in nodal and internodal parameters. Biophys J.

[CR42] Nagumo J, Arimoto S, Yoshizawa S (1962). An active pulse transmission line simulating nerve axon. Proc IRE.

[CR43] Neishabouri A, Faisal A (2014). Saltatory conduction in unmyelinated axons: clustering of Na+ channels on lipid rafts enables micro-saltatory conduction in C-fibers. Front Neuroanat.

[CR44] Pakdaman K, Thieullen M, Wainrib G (2010). Fluid limit theorems for stochastic hybrid systems with applications to neuron models. Adv Appl Probab.

[CR45] Pines D (1999). Elementary excitations in solids.

[CR46] Pitarke JM, Silkin VM, Chulkov EV, Echenique PM (2007). Theory of surface plasmons and surface-plasmon polaritons. Rep Prog Phys.

[CR47] Rall W, Kandel E (1977). Core conductor theory and cable properties of neurons. Handbook of physiology, Section 1, the nervous system, part 1.

[CR48] Rall W, Koch C, Segev I (1989). Cable theory for dendritic neurons. Methods in neuronal modeling: from synapses to networks.

[CR49] Rall W, Burke R, Holmes W, Jack J, Redman S, Segev I (1992). Matching dendritic neuron models to experimental data. Physiol Rev.

[CR50] Richardson AG, Mclntyre C, Grill W (2000). Modelling the effects of electric fields on nerve fibres: influence of the myelin sheath. Med Biol Eng Comput.

[CR51] Scurfield A, Latimer DC (2018). A computational study of the impact of inhomogeneous internodal lengths on conduction velocity in myelinated neurons. PLoS ONE.

[CR52] Sommerfeld A (1899). Über die fortpflanzung elektrodynamischer Wellen langs eines Drahts. Annalen der Physik und Chemie.

[CR53] Song X, Wang H, Chen Y, Lai Y-C (2019). Emergence of an optimal temperature in action-potential propagation through myelinated axons. Phys Rev E.

[CR54] Thomson W (1854). On the theory of the electric telegraph. Proc R Soc Lond.

[CR55] Waxman S, Bennett M (1972). Relative conduction velocities of small myelinated and non-myelinated fibres in the central nervous system. Nat New Biol.

[CR56] Zayats AV, Smolyaninov II, Maradudin AA (2005). Nano-optics of surface plasmon polaritons. Phys Rep.

[CR57] Zhao LL, Kelly KL, Schatz GC (2003). The extinction spectra of silver nanoparticle arrays: influence of array structure on plasmon resonance wavelength and width. J Phys Chem B.

[CR58] Zou S, Janel N, Schatz GC (2004). Silver nanoparticle array structures that produce remarkably narrow plasmon lineshapes. J Chem Phys.

