# Supplementary material for: New wave-type mechanism of saltatory conduction in myelinated axons and micro-saltatory conduction in C fibres
Source: Eur Biophys J. 2020 Jun 25;49(5):343–60. doi: 10.1007/s00249-020-01442-z (PMC7351862; doi:10.1007/s00249-020-01442-z)
Supplement: Supplementary file 1 — Supplementary Material [file 249_2020_1442_MOESM1_ESM.pdf]

Supplementary Information

A Cable model as the diffusion-like limit of the transmission long line

The long line (like a coaxial line, particularly well corresponding to a bare neuron cell cord separated by a thin insulator membrane from the outer electrolyte of inter-cellular cytosol) is assumed to be a series of infinitely short segments of length dx, represented by the scheme in Fig. 10. R is the longitudinal resistance of the inner line per length unit, L is the distributed inductance of the inner wire per length unit, C is the capacitance between two line components across the separating dielectric layer also per unit length of the line, G is the electrical conductivity across the barrier per unit length of the line.

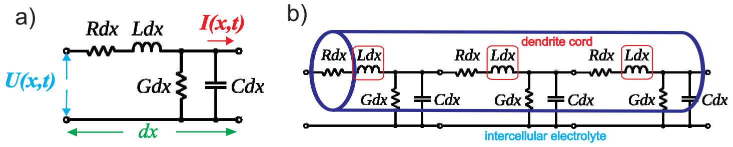

Fig. 10 a) Elementary segment of the transmission line of length dx. R and L are resistance and inductance of the inner wire (the upper line in the figure) per length unit, C and G are the capacity and conductivity across the insulating barrier per unit of the length of the line. The electrical current flows only along the inner wire. b) Transmission line applied to a dendrite—the inner wire is the dendrite cord (with $L = 0$) and outer coaxial screen is the intercellular electrolyte separated by the dendrite (or unmyelinated axon) cell membrane.

Due to Ohm's law and the definition of the capacitance and the inductance one gets the relations between the voltage gated line segment and the current along the inner wire,

$$\begin{aligned} \frac{\partial U(x,t)}{\partial x} &= -L\frac{\partial I(x,t)}{\partial t} - RI(x,t), \\ \frac{\partial I(x,t)}{\partial x} &= -C\frac{\partial U(x,t)}{\partial t} - GU(x,t), \end{aligned} \tag{11}$$

which are two coupled differential equations for the complex voltage V and current I. Taking the derivative $\frac{\partial}{\partial x}$ of both equations and substituting one into another, we can arrive at the second order differential equations,

$$\begin{aligned} \frac{\partial^2 U(x,t)}{\partial x^2} - LC\frac{\partial^2 U(x,t)}{\partial t^2} &= (RC+GL)\frac{\partial U(x,t)}{\partial t} + GRU(x,t), \\ \frac{\partial^2 I(x,t)}{\partial x^2} - LC\frac{\partial^2 I(x,t)}{\partial t^2} &= (RC+GL)\frac{\partial I(x,t)}{\partial t} + GRI(x,t). \end{aligned} \tag{12}$$

One can notice that both equations ate identical (they are conventionally called as telegrapher's equations). For the lossless case, i.e., when $R = G = 0$, Eqs (12) gain the form of the wave equations both for U and I, describing the ideal wave-type transmission along x direction with the velocity $v = \frac{1}{\sqrt{LC}}$ and without any losses. This corresponds to an ideal coaxial line. This is, however, not the case for a neuron, for which the assumption $L = 0$ is appropriate. Then Eqs. (12) attain the form of 1D diffusion equation,

$$\begin{aligned} \frac{\partial^2 U(x,t)}{\partial x^2} &= RC\frac{\partial U(x,t)}{\partial t} + GRU(x,t), \\ \frac{\partial^2 I(x,t)}{\partial x^2} &= RC\frac{\partial I(x,t)}{\partial t} + GRI(x,t). \end{aligned} \tag{13}$$

Defining the parameters $\lambda = \frac{1}{\sqrt{GR}}$ and $\tau = \frac{C}{G}$, one can rewrite the above equation (for U) in a conventional form,

$$\lambda^2 \frac{\partial^2 U(x,t)}{\partial x^2} = \tau\frac{\partial U(x,t)}{\partial t} + U(x,t). \tag{14}$$

The parameter λ defines the spatial scale of the diffusion, whereas τ is its time scale. The velocity of the diffusion of the signal, i.e., of the diffusive current along the dendrites (or unmyelinated axon) is assumed as $v_c = \frac{\lambda}{\tau} = \frac{\sqrt{G}}{C\sqrt{R}}$. This velocity is the larger the smaller C and R and the larger G are. The range of the diffusion, $\sim \lambda$, lowers, however, with growing G (due to the shunt escape of the current). Larger G results in larger velocity but severely limits the range. The overall behavior of the $U(x,t)$ (or I) diffusion defined by Eq. (14) is illustrated in Fig. 11, which presents the solution of Eq. (14) for initial condition in the form of periodic excitation in $x = 0$ point, $U(0,t) = U_0 cos(t)$. One can notice that the cable theory (i.e., Eq. (14)) gives the non-wave-type propagation related to an ordinary current (and voltage signal) of diffusion type, thus on a relatively short distance with a quickly lowering amplitude. For the realistic values of R, C, G and d in axons, the estimation of v_c gives $0.05 - 1$ m/s. This is too low for explanation of the saltatory conduction in myelinated axons.

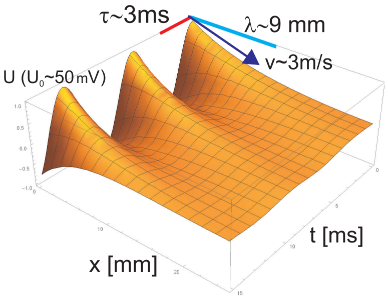

Fig. 11 The solution of Eq. (14) for $\lambda = 9$ mm and $\tau = 3$ ms and the ignition signal at $x = 0$ point, $U(0,t) = 50cos(t)$ mV. The unit proportions in the figure are conserved.

The observed conduction velocity of a myelinated axon is by a factor of $100 - 200$ greater than that of a geometrically similar unmyelinated axon. Myelinated neurons make up a large proportion of all neurons in the human body, more so in the CNS. Specifically, all PNS neurons with diameters greater than around 1 μm and all CNS neurons with diameters greater than around 0.2 μm are myelinated [37–39]. In a study of myelinated axons in the human brain, most were found to have a diameter less than 1 μm. The cable theory applied to myelinated axons gives at most 6 times larger velocity and to consider a velocity ~ 40 m/s the axon diameter and internodal length assumed are extremely large [34] in contrast to observations [37, 38, 34]. Data indicate that the v_m is roughly proportional to fiber diameter, d, but this scale factor does vary. The complex factors impacting the v_m in myelinated axons can be found in [37, 39] based on the cable theory variants, including, for instance, myelin thickness and capacitance (taking into account that the cable theory speed v_c scales as $\sqrt{d}$). In the plasmon-polariton model we have got $v_m \sim d$.
